# Role of the International Journal of Cardiovascular Sciences in the Increase of Brazilian publications in Cardiology and Cardiovascular Sciences

**DOI:** 10.5935/abc.20160109

**Published:** 2016-07

**Authors:** Luiz Felipe P. Moreira

**Affiliations:** Instituto do Coração - Hospital das Clínicas - Faculdade de Medicina - Universidade de São Paulo (USP), São Paulo, SP - Brazil

**Keywords:** Cardiovascular Diseases, Prevalence, Periodicals as Topic

The number of Brazilian scientific publications in the field of Cardiology and Cardiovascular Sciences in international indexing journals, different from other areas of Medicine, has remained largely stable over the past eight years. This situation does not reflect the significant increase in the number of senior researchers and the number of studies carried out in the several fields of activity that constitute our medical specialty. We currently observe the development of our Postgraduate Programs, as well as the opening of new research centers focused on the study of cardiovascular diseases in several universities of the country.

On the other hand, in spite of the increased number of international journals in our area, with the creation of "families of journals" associated to the periodicals of highest significance, the publication of Brazilian articles of greater impact on the area of cardiology remains limited. Additionally, we have not had the incorporation of new journals published in Brazil in the field of cardiovascular science, which prevents the access of Brazilian researchers to the scientific dissemination of their work. The *Arquivos Brasileiros de Cardiologia*, the journal responsible for the disclosure of approximately 20% of national publications indexed in our area of operation, can now publish only 25% of the submitted articles, of which approximately 10% comes from foreign research centers.

The selective publication policy of the *Arquivos Brasileiros de Cardiologia* tends to gradually increase its impact factor, which has varied between 1 and 1.2 in the past five years, according to data released by the Journal of Citation Report of Thompson Reuters. Similarly, the numbers achieved by our publications, of which progressive increase is now reaching the average numbers similar to countries such as the United States, Mexico and South Korea, indicate the same direction. Undoubtedly, the best qualification of journals published in Brazil meets the interests of the postgraduate programs of our country, which need a journal with the best score at the international level for the dissemination of studies arising from dissertations and doctoral theses. On the other hand, the occurrence of this fact limits the adequate dissemination of studies carried out by many Brazilian researchers, due to the difficulty of having access to other means of publication.

Based on these facts, the Brazilian Society of Cardiology (SBC) has decided to increase its activities in the field of scientific publications, by incorporating a new journal into its portfolio. Starting this month, the International Journal of Cardiovascular Sciences (IJCS), a bimonthly publication that was conducted by the Cardiology Society of the State of Rio de Janeiro, has started to be conducted by our society. With this step, SBC aims to broaden the perspective and scope of the Brazilian cardiology science, the impact of which has been recognized by several international and foreign societies, as evidenced by the several studies accepted for presentation at the most important conferences and scientific meetings of our medical specialty.

IJCS aims to increase the number of articles published internationally, working as a means of dissemination not only of the Brazilian cardiology science, but also at the integration of Brazilian research with research centers in other countries. The journal is now in its second year of publication, currently going to be indexed by SciELO, a situation that guarantees visibility in other international databases, such as Web of Science and ScienceOpen. While maintaining its periodicity and the incorporation of the same editorial quality of the Brazilian Archives of Cardiology, the new journal should attain indexing in PubMed and also in the Scopus system within one year.

The expansion of the publishing activities of SBC and the incorporation of IJCS must be complemented by the creation of a portal for specialized scientific journals in the area of cardiovascular sciences. This proposal has as its main justification to encourage interaction among journals published in Brazil in our area, facilitating the publication of editorials, letters and review articles. This promotes the elevation of the impact factors of all involved journals and should result in the effective creation of a virtuous circle for the Brazilian cardiology science.

